# P33 - Causes of anaphylaxis in pediatric population

**DOI:** 10.1186/2045-7022-4-S1-P88

**Published:** 2014-02-28

**Authors:** Maria Jose Castillo, Marta Viñas, Nora Hernández, Marcel Ibero

**Affiliations:** 1 Terrassa's Hospital, Terrassa, Spain

## Objective

To determine the number of early diagnoses of anaphylaxis and its causes during the years 2011 and 2012 in children up to 14 years-old.

## Material and methods

We performed a retrospective study of all registered diagnoses of anaphylaxis in our unit between 2011 and 2012 in children up to the age of 14. We included all patients who had anaphylaxis in these years, both new patients and follow-up visits.

## Results

We found 41 patients in total, with an average age of 5 years and 11 months (2 months-14 years). 27 (66%) patients were boys and 14 (34%) patients were girls. In all cases of anaphylaxis, symptoms were due to one or several foods except one, which was due to an antibiotic (amoxicillin). The foods involved were nuts (15,36.6%), egg (8,19.5%), fruits (5,12.2%), LTP syndrome (3,7.3%), cow's milk proteins, vegetables and seafood (2,4.9%) and cereals, fish, goat and sheep milk, potatoes and mushrooms (1, 2.4%).When we reviewed the causes of anaphylaxis by age-group we found that in children under three years the most frequent cause was the egg followed by nuts, in children 3 to 6 years the most frequent cause were nuts and in children 6 to 14 years the causes were more varied. In 18 patients (43%) other allergic diseases were present (food allergy, respiratory allergy, atopic dermatitis and sensitizations without clinical symptoms). The most frequent symptoms were cutaneous symptoms associated with gastrointestinal clinic.

## Conclusion

The most frequent causes of anaphylaxis in our unit in the pediatric age are nuts and egg. The appearance of anaphylaxis is higher in children younger than 3 years and in males.

